# Measuring multimorbidity in a working population: the effect on incident sickness absence

**DOI:** 10.1007/s00420-015-1104-4

**Published:** 2015-11-28

**Authors:** Monica Ubalde-Lopez, George L. Delclos, Fernando G. Benavides, Eva Calvo-Bonacho, David Gimeno

**Affiliations:** CISAL-Center for Research in Occupational Health, Universitat Pompeu Fabra, Av Dr Aiguader, 88, PRBB building, 1st floor, Barcelona, Spain; CIBERESP, CIBER in Epidemiology and Public Health, Madrid, Spain; IMIM (Institut Hospital del Mar Medical Research Institute), Barcelona, Spain; Southwest Center for Occupational and Environmental Health, Department of Epidemiology, Human Genetics, and Environmental Sciences, The University of Texas School of Public Health, Houston, TX USA; Ibermutuamur (Mutua de Accidentes de Trabajo y Enfermedades Profesionales de la Seguridad Social 274), Madrid, Spain; Southwest Center for Occupational and Environmental Health, Department of Epidemiology, Human Genetics, and Environmental Sciences, The University of Texas School of Public Health, San Antonio Campus, San Antonio, TX USA

**Keywords:** Health conditions, Longitudinal, Occupational health, Sick leave

## Abstract

**Purpose:**

Multimorbidity research typically focuses on chronic and common diseases in patient and/or older populations. We propose a multidimensional multimorbidity score (MDMS) which incorporates chronic conditions, symptoms, and health behaviors for use in younger, presumably healthier, working populations.

**Methods:**

Cross-sectional study of 372,370 Spanish workers who underwent a standardized medical evaluation in 2006. We computed a MDMS (range 0–100) based on the sex-specific results of a multicorrespondence analysis (MCA). We then used Cox regression models to assess the predictive validity of this MDMS on incident sickness absence (SA) episodes.

**Results:**

Two dimensions in the MCA explained about 80 % of the variability in both sexes: (1) chronic cardiovascular conditions and health behaviors, and (2) pain symptoms, in addition to sleep disturbances in women. More men than women had at least one condition (40 vs 15 %) and two or more (i.e., multimorbidity) (12 vs 2 %). The MDMS among those with multimorbidity ranged from 16.8 (SD 2.4) to 51.7 (SD 9.9) in men and 18.5 (SD 5.8) to 43.8 (SD 7.8) in women. We found that the greater the number of health conditions, the higher the risk of SA. A higher MDMS was also a risk factor for incident SA, even after adjusting for prior SA and other covariates. In women, this trend was less evident.

**Conclusions:**

A score incorporating chronic health conditions, behaviors, and symptoms provides a more holistic approach to multimorbidity and may be useful for defining health status in working populations and for predicting key occupational outcomes.

**Electronic supplementary material:**

The online version of this article (doi:10.1007/s00420-015-1104-4) contains supplementary material, which is available to authorized users.

## Background

As the world’s population ages, the prevalence of multiple chronic and non-chronic health-related conditions is increasing (Fortin et al. 2007). One in four adults has at least two chronic conditions, more than half of older adults has three or more, and most primary care patients have coexisting conditions (Boyd et al. 2007). And, while we live longer as a result of advances in clinical care and public health policies, we are also working longer due to increases in the retirement age and living with coexisting health conditions and unhealthy behaviors affecting not only our quality of life, but also our ability to work, employability, and disability (Robson et al. 2001; Fortin et al. 2004; Bevan et al. 2009).

The coexistence of two or more health conditions has been, for some time, referred to indistinctly as *comorbidity*, i.e., the occurrence of any additional condition affecting the course and treatment of a primary condition (Feinstein 1970; Valderas et al. 2009), or *multimorbidity*, i.e., the occurrence of two or more chronic conditions with none considered the primary condition (van den Akker et al. 1998). During the last decade, rather than focusing on single pathologies, patterns of medical care are evolving toward a more holistic approach with increasing interest in the epidemiology of multimorbidity (Fortin et al. 2007; Starfield 2011a).

To date, however, indicators of multimorbidity have largely been constructed on the basis of chronic health conditions or common diseases and mainly focused on patient and/or older populations. The applicability of these indicators of multimorbidity to other younger and healthier populations, such as the working population (Li and Sung 1999), has not been well studied. In the workforce, however, chronic diseases might be not as prevalent as in commonly studied populations, while certain unhealthy behaviors that are risk factors for later development of chronic health conditions are (Miller 2011). Health risk factors raise the probability of adverse health outcomes (i.e., mortality, disability and chronic conditions) (WHO 2009), but whether those risk factors impact the clustering of chronic conditions is an incipient idea to be further explored. It is expected that, by 2020, the proportion of the workforce age 50 and over will increase substantially, as will the number and range of morbidities (Great Britain. Department for Work and Pensions 2009). Identifying and improving health behaviors earlier could reduce the onset of future morbidity, leading to better health status and lower disease burden in later ages.

The adverse impact of multiple chronic conditions on occupational outcomes, such as sickness absence (SA) or work ability has been previously described (Koolhaas et al. 2014; Casimirri et al. 2014). However, individuals, usually older workers, were grouped based simply on the presence or absence, number, or combinations of chronic conditions (Kessler et al. 2001; Collins et al. 2005).

Regarding its measurement, there is a lack of uniformity in the operationalization of multimorbidity (Fortin et al. 2012), with great heterogeneity both in the selection (i.e., frequently, chronic conditions are selected based on the highest prevalence and/or mortality rates in the study population (Diederichs et al. 2011) which varies by default) and number (i.e., coexisting diseases may be simply counted, ranging from 6 to over 100) of chronic conditions chosen (Huntley et al. 2012). In addition, multimorbidity indices are usually intended to predict specific outcomes.

Some calculate risk of death based on age and mortality rates of comorbid conditions (e.g., Charlson Comorbidity Index) (D’Hoore et al. 1996) or hospitalization rates based on pharmacy data (e.g., Chronic Disease Score) (Von Korff et al. 1992), while others calculate physical impairment (e.g., Functional Comorbidity Index) (Groll et al. 2005) or health status (e.g., KoMo score) (Glattacker et al. 2007) based on disease severity. Standardized indices may facilitate comparability, but the focus on specific predefined diseases and outcomes limits their generalizability and assumes these diseases and related predictive effects are the ones of interest, disregarding the potential impact of multimorbidity on other outcomes. In addition, these indices have a priori assigned weighting schemes that adjusted for severity of condition but which may need to be updated, as the index–outcome relationship may change over time. Given all the above, while these indices may be useful for the specific outcome they are designed to capture, they may be of limited use to reflect the effect of multimorbidity on a given population as a whole.

To overcome these restraints, we propose calculating a multidimensional multimorbidity score (MDMS) based on examining the relationship between health-related conditions, available in many population databases, without initially considering its impact on a specific outcome. Further, individuals living with multimorbidity may cope well and without any intervention, whereas others may not, due to other health-related factors. To better reflect this complex scope, the common clinical concept of multimorbidity may be expanded by going beyond chronic diseases, examining how they overlap at specific points in time with other health-related conditions, risk factors, health behaviors, or even psychological distress (Mercer et al. 2009). To our knowledge, few studies have looked into the clustering of chronic health conditions (Prados-Torres et al. 2014; Garin et al. 2014), even fewer in groups healthier than the general population, such as the working population (Holden et al. 2011), and none including other health-related conditions beyond chronic diseases. Such a score could be useful for determining the burden and distribution of multimorbidity in a working population, and by extension its health status, as well as to predict target occupational outcomes.

## Methods

The study population consisted of 372,370 workers registered with the Spanish social security system and covered by one of the largest state health mutual insurance companies (*mutua*). These workers underwent a standardized medical evaluation in 2006 by a subsidiary company focused on illness and injury prevention (“prevention service”). The study proposal was reviewed and approved by the Clinical Research Ethics Committee of the Parc de Salut Mar in Barcelona, and an agreement assuring participant confidentiality was signed by all stakeholders. Data were treated confidentially in accordance with current Spanish legislation on data protection. All data were de-identified before being delivered to the research team. All participants gave informed consent for their data to be included in the study.

Each evaluation was performed by an occupational physician, and included completion of a uniform questionnaire and measurement of body mass index (BMI) as part of the physical examination. The questionnaire included demographic, labor, and clinical variables and had been developed by the mutua’s occupational health service personnel (technicians, researchers, and occupational physicians) for general health surveillance purposes.

Basic socio-demographic and labor characteristics included sex (female/male), age (grouped as <25, 25–34, 35–44, 45–54, 55–65, >65 years), and occupation coded using the Spanish National Classification of Occupation (CNO93) and grouped by occupational social class (Regidor 2001) [I–management (≥10 employees), II–management (<10 employees), IIIa–administrative, IIIc–manual workers’ supervisor, IVa–skilled manual workers, IVb–semi-skilled manual workers, and V–unskilled workers]. The questionnaire collected data on prior diagnoses of chronic conditions (hypertension, hyperlipidemia, diabetes, venous thrombosis, coronary artery disease, cerebrovascular disease, and/or peripheral vascular disease), health behaviors (tobacco and alcohol consumption), and selected symptoms (headache, fatigue, sleep disturbances, neck and low back pain). Questions on chronic conditions and symptoms were formulated as Yes or No, whereas sleep disturbances were categorized as “able to sleep continuously more than 6 h,” “sleep is disrupted during the night,” and “sleep is disrupted in the early morning.” Tobacco use was classified as never, current or ex-smoker, and alcohol consumption as never drinker, occasional (less than once a week), weekend, daily moderate [<140 g of alcohol weekly, daily high (equal or more than 140 g of alcohol weekly)], and former drinker.

Construction of the MDMS was developed in two steps. First, we ran a multiple correspondence analysis (MCA) with the joint method (JCA) including the eight previously described chronic conditions; the five symptoms, tobacco and alcohol consumption. The MCA is a data analysis technique used to identify patterns of relationships between more than two sets of categorical variables by using multiway cross-tabulation (Abdi and Valentin 2007). Two key parameters are provided by the MCA: inertia (i.e., percentage of explained variance for each dimension or axis obtained) and the contribution of the variables’ categories (i.e., absolute, or the inertia relative to the principal inertia on an axis; and relative, or the inertia relative to the inertia of a category) (Greenacre 2003). The addition of all absolute contributions is 1 for a given dimension, which allows the identification of the most relevant categories. The closer the relative contribution of a given category to 1, the better is it represented within the dimension. Those categories contributing the most to the inertia of each dimension (absolute contribution) and those better represented within the dimension (i.e., relative contribution closer to 1) will be considered relevant. The JCA method of the MCA corrects the percentages of the explained variance obtained with MCA and can be interpreted as a factor analytic model or a generalization of principal component analysis (Greenacre 1984).

All analyses were conducted for men and women separately. While the variables, obtained from the standardized questionnaire, initially included in the MCA were the same for men and women, we conducted sex-specific analysis based on the following considerations: (1) since males represented 70 % of the sample, calculating sex-specific multimorbidity scores helped avoid overall effect attributions; (2) the prevalence of specific chronic health conditions was different for both sexes, which in turn would lead to different multimorbidity prevalence as the MDMS is based on relationships among health-related conditions; and (3) the combination of significant health-related conditions accounting for the two dimensions obtained, in addition to their absolute contributions (weights), differed by sex.

In a second step, we developed an algorithm based on the contributions of the categories for each of the variables weighting significantly in the dimensions obtained from the JCA. Of all 15 variables, with 37 categories overall, those showing an absolute contribution equal to or greater than the mean absolute contribution of all variables included in the JCA (i.e., 1/15) were considered in the calculation of the dimension score. Within these variables, we selected those categories with an absolute contribution equal to or greater than the mean absolute contribution of the corresponding variable, and a relative contribution ≥0.3. The final MDMS was the sum of the value for the weighted absolute contributions (i.e., dimensions scores *x* inertia) of each dimension obtained. The algorithm applied was MDMS = [(ScD1 + ScD2)/max ScD] × 100 = {[(Σ AbsC × InertiaD1) + (Σ AbsC × InertiaD2)]/max ScD} × 100, where multidimensional multimorbidity score is the MDMS; Sc is the score for each dimension; D1 and D2 are the first and second dimensions, respectively; AbsC refers to the categories’ absolute contribution, and max ScD is the maximum score for each sex. The MDMS ranged from zero (no multimorbidity) to 100 (high multimorbidity). For example, a man with obesity (AbsC = 0.065), diabetes (AbsC = 0.081), and headache (AbsC = 0.072) would have a low MDMS level of 18.4, calculated as follows: [(0.065 + 0.081) × 0.65 + (0.072 × 0.18)]/0.586} × 100, where 0.586 is the max ScD calculated for men.

Additionally, we calculated two alternative MDMSs by including: (a) chronic conditions alone, and (b) chronic conditions and symptoms, in order to compare its distribution with our proposed MDMS. Individuals were categorized with none, one, two, and more than two self-reported health conditions. Multimorbidity was considered present when there were at least two co-occurring health conditions. Among persons with multimorbidity, the MDMS was grouped into tertiles (low, medium, and high multimorbidity).

In a final step, the MDMS, together with SA occurring in the 2 years prior to the 2006 medical evaluation, were used to fit Cox models, adjusted by age and occupational social class, to test its ability to predict new first SA episodes, expressed as the crude (HRc) and adjusted (HRa) hazard ratios and corresponding 95 % confidence intervals (95 % CI). Information on prior SA episodes, occurring during the 2 years prior to medical evaluation, incident SA and other socio-demographic variables, were obtained from the social security data system, which is the official registry for SA episodes in Spain (Benavides et al. 2014).

**Fig. 1:**
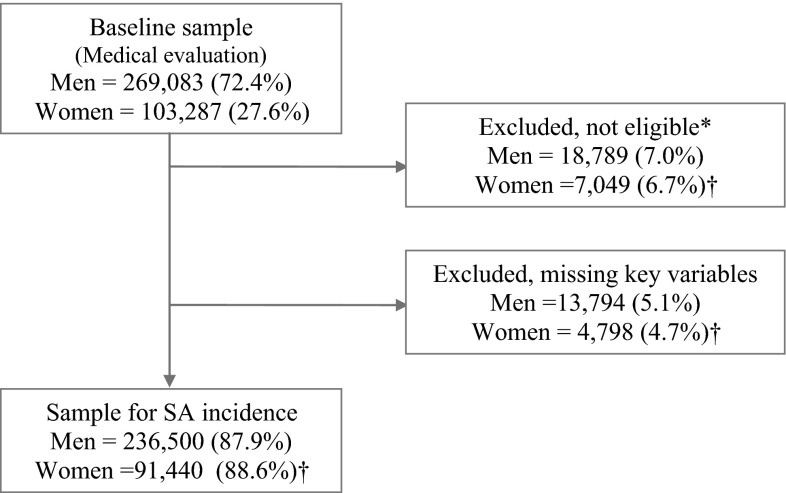
Flowchart of sample selection. *Persons whose coverage by the mutual insurance company ended prior to the 2006 medical evaluation or before a recorded SA episode, and those who were on SA leave during the medical evaluation. ^†^Percentage refers to the figures showed in the preceding box

The final sample for the survival models (236,500 men and 91,440 women) excluded individuals on sick leave during the medical evaluation, those lacking insurance coverage before the medical evaluation or the new SA episode, and those with missing data on key variables (Fig. 1). Statistical analyses were performed using Stata/MP v. 13 ©.

## Results

Of the overall insured population in 2006 (annual average, 980,463 workers), about 45 % of men and 28 % of women underwent a medical evaluation for a total sample of 372,370 individuals (72 % men). In both sexes, the most prevalent age group was the 25–34 year group [mean, 35.9 years (SD 9.8) for women; 37.9 years (SD 11.2) for men], confirming the generally young age distribution. Administrative and skilled manual were the most prevalent occupational social classes for women and men, respectively. Hyperlipidemia and hypertension were the most prevalent chronic conditions, and men were more likely to be overweight or obese. Regarding health behaviors, 44 % of men and 37 % of women were current smokers, and 53 % of women and 28 % of men reported consuming no alcohol (Web Appendix 1). The total symptom prevalence was 14 % for men and 26 % for women; of these, sleep disturbances at night (42 vs 40 %), fatigue (10 vs 17 %), low back pain (13 vs 9 %), and neck pain (6 vs 12 %) were the most common, in addition to headache for women (4.6 %) (Web Appendix 2).

From the MCA analysis, for both men (*n* = 222,506) and women (*n* = 84,113) with at least one health condition, we obtained two dimensions that explained 83 and 77 % of the total variability, respectively. The first dimension (D1) was related to cardiovascular conditions and health behaviors, and the second (D2) included symptoms. In men, D1 (65 % of inertia) was composed of the following categories: hyperlipidemia, hypertension, diabetes, coronary artery disease, obesity, and being a former smoker. In women, D1 (49 % of inertia) included venous thrombosis, coronary artery disease, cerebrovascular disease, and peripheral vascular disease. In men, D2 (18 % of inertia) included headache, neck, and low back pain, and in women, D2 (28 % of inertia) included these same symptoms, in addition to sleep disruption at night (Table 1). While only relevant categories from the MCA results are shown in Table 1, the nonsignificant categories are listed in the table footnote. As previously mentioned, relative contributions reflect how well categories are represented in a dimension (i.e., values closer to 1 indicate a better representation of the category within a dimension) taking into account a given variable is considered well represented in a dimension if the relative contribution of the variable is not <0.6 (or 60 %), or analogously, its categories’ relative contribution is at least 0.3 (or 30 %) (Greenacre 1984).

**Table 1:** Multiple correspondence analysis (MCA) results according to the contributing variables

	Total inertia	Dimension inertia	Category inertia	Variable absolute contribution	Category relative contribution	Mean absolute contribution	Category absolute contribution
Men (*n* = 222,506)^a^	0.830						
Dimension 1		0.650					
Hyperlipidemia			0.114	0.186	0.972	0.09	0.171
Hypertension			0.184	0.297	0.975	0.15	0.278
Diabetes			0.056	0.083	0.936	0.04	0.081
Coronary artery disease			0.055	0.072	0.855	0.04	0.072
Obesity (BMI > 30)			0.047	0.131	0.897	0.03	0.065
Former smoker			0.058	0.100	0.887	0.03	0.079
Dimension 2		0.180					
Headache			0.015	0.072	0.877	0.04	0.072
Low back pain			0.063	0.344	0.951	0.17	0.338
Neck pain			0.083	0.448	0.947	0.22	0.444
Women (*n* = 84,113)^b^	0.770						
Dimension 1		0.490					
Venous thrombosis			0.078	0.144	0.909	0.07	0.140
Coronary artery disease			0.081	0.154	0.936	0.08	0.154
Cerebrovascular disease			0.090	0.161	0.884	0.08	0.161
Peripheral vascular disease			0.174	0.305	0.862	0.15	0.305
Dimension 2		0.280					
Headache			0.028	0.084	0.846	0.04	0.084
Low back pain			0.110	0.332	0.856	0.17	0.332
Neck pain			0.112	0.347	0.860	0.17	0.337
Sleep disturbances			0.034	0.069	0.569	0.02	0.046

^a^Men with any of health conditions included in the MCA. Nonsignificant conditions: venous thrombosis, cerebrovascular disease, peripheral vascular disease, fatigue, alcohol consumption, sleep disturbances

^b^Women with any of health conditions included in the MCA. Nonsignificant conditions: hypertension, hyperlipidemia, diabetes, BMI, fatigue, tobacco and alcohol consumption

Table 2 shows the distribution of the MDMS according to three different groups: based solely on chronic conditions, chronic conditions plus symptoms, or chronic conditions plus both symptoms and habits. For both sexes, the proportion of individuals identified as having morbidity or multimorbidity was greater, and mean scores by number of conditions lower, when all three health conditions were used. In this latter group, 40 % of men and 15 % of women reported at least one of these conditions.

**Table 2:** Distribution of the multidimensional multimorbidity score (MDMS) by type and number of health conditions included

Number	Men (*n* = 269,083)		Women (*n* = 103,287)	
	Score mean (SD)	*n* (%)	Score mean (SD)	*n* (%)
Chronic diseases, symptoms, habits^a^				
0	0 (–)	160,905 (59.8)	0 (–)	87,655 (84.9)
1	11.1 (6.5)	74,824 (27.8)	6.9 (7.1)	13,607 (13.2)
2	27.1 (11.0)	24,466 (9.1)	26 (9.8)	1671 (1.6)
>2	50.6 (13.3)	8888 (3.3)	40.5 (9.8)	354 (0.3)
Chronic diseases, symptoms^b^				
0	0 (–)	188,417 (70)	0 (–)	98,080 (95)
1	11.7 (8.5)	61,571 (22.9)	27.4 (3.5)	4376 (4.3)
2	32.6 (10.8)	14,765 (5.5)	55.3 (2.9)	815 (0.8)
>2	52.8 (10.4)	4330 (1.6)	77.0 (11)	16 (0.1)
Chronic diseases^c^				
0	0 (–)	233,548 (86.8)	0 (–)	99,910 (96.8)
1	39.0 (14.0)	30,171 (11.3)	43.0 (4.2)	3330 (3.3)
2	39.0 (14.0)	5221 (2)	61.8 (6.4)	39 (0.1)
>2	81.3 (7.6)	143 (0.1)	100.0 (–)	8 (0.1)

^a^Men: hyperlipidemia, hypertension, diabetes, coronary artery disease, obesity, former smoker, headache, low back pain, neck pain. Women: venous thrombosis, coronary artery disease, cerebrovascular disease, peripheral vascular disease, headache, low back pain, neck pain, sleep disturbances

^b^Men: hyperlipidemia, hypertension, diabetes, coronary artery disease, obesity, headache, low back pain, neck pain. Women: venous thrombosis, coronary artery disease, cerebrovascular disease, peripheral vascular disease, low back pain, neck pain

^c^Men: hyperlipidemia, hypertension, venous thrombosis, cerebrovascular disease, peripheral vascular disease. Women: hypertension, coronary artery disease, peripheral vascular disease

Among individuals with multimorbidity (12 % of men; 2 % of women), the overall mean score was 33 (SD 16) for men and 28 (SD 11) for women. Half of men and women showed a score lower than 29 and 25, respectively (Table 3).

**Table 3:** Distribution of the multidimensional multimorbidity score (MDMS) among men and women with ≥2 health conditions

MDMS	Score	*n* (%)
Men (*n* = 33,354)		
Range	9–100	–
Mean (SD)^a^	32.9 (15.5)	–
P50 (P25–P75)^b^	28 (16–40)	–
MDMS levels [mean (SD)]^a^		
Low MDMS (first tertile, ≤25 points)	16.8 (2.4)	11,340 (34.0)
Medium MDMS (second tertile, >25 to ≤38 points)	31.4 (5.0)	11,538 (34.6)
High MDMS (third tertile, >38 points)	51.7 (9.9)	10,476 (31.4)
Women (*n* = 2025)		
Range	7–100	–
Mean (SD)^a^	28.1 (11.2)	–
P50 (P25–P75)^b^	24 (21–38)	–
MDMS levels [mean (SD)]^a^		
Low MDMS (first tertile, ≤22 points)	18.5 (5.8)	908 (44.8)
Medium MDMS (second tertile, >22 to ≤38 points)	33.3 (6.4)	834 (41.2)
High MDMS (third tertile, >38 points)	43.8 (7.8)	283 (14.0)

^a^SD: standard deviation

^b^P50: median value (50th percentile); P25: 25th percentile; P75: 75th percentile

The final analytical sample used in the Cox models showed no significant differences across MDMS levels with those excluded from analysis (men, *p* = 0.11; women, *p* = 0.84). In both sexes, both prior SA episodes and an increasing number of health conditions were associated with a greater risk of SA incidence. A trend toward higher risk of new SA episodes was observed among men as MDMS levels increased; from HRa = 1.04 (95 % CI 1.01–1.08) when there was one health condition present to HRa = 1.20 (95 % CI 1.12–1.28) at high MDMS levels. Women showed a similar trend, although the HRa values did not reach statistical significance. When stratified by prior SA episodes and other covariates, this effect persisted (Table 4).

**Table 4:** Associations (HR) of levels of multidimensional multimorbidity score (MDMS) and number of previous sickness absence (SA) episodes with total incident SA episodes, during 2 years of follow-up after medical evaluation in 2006, in men and women

	Total			Previous SA		No previous SA	
	(*n* = 236,500)			(*n* = 14,714)		(*n* = 221,786)	
	Cases	HRc (95 % CI)^a^	HRa (95 % CI)^b^	Cases	HRa (95 % CI)^b^	Cases	HRa (95 % CI)^b^
Men							
Morbidity	17,193			9998		7195	
No health condition	8749	1.00	1.00	4982	1.00	3767	1.00
One health condition	5218	1.27 (1.22–1.31)	1.04 (1.01–1.08)	3051	0.98 (0.94–1.03)	2167	1.12 (1.06–1.18)
≥2 health conditions							
Low MDMS (first tertile, ≤25 points)	1014	1.62 (1.52–1.73)	1.14 (1.06–1.21)	604	1.01 (0.93–1.10)	410	1.33 (1.2–1.48)
Medium MDMS (second tertile, >25 to ≤38 points)	1057	1.65 (1.55–1.76)	1.10 (1.03–1.18)	643	0.99 (0.91–1.08)	414	1.30 (1.17–1.44)
High MDMS (third tertile, >38 points)	1155	1.98 (1.86–2.11)	1.20 (1.12–1.28)	718	1.07 (0.98–1.16)	437	1.45 (1.31–1.62)
Previous SA							
No episodes	7195	1	1		–		–
1–4 episodes	9735	1.92 (1.91–1.93)	1.89 (1.87–1.90)		–		–
>4 episodes	263	2.20 (2.14–2.25)	2.16 (2.11–2.22)		–		–

	Total			Previous SA		No previous SA	
	(*n* = 91,440)			(*n* = 6303)		(*n* = 87,137)	
	Cases	HRc (95 % CI)^a^	HRa (95 % CI)^b^	Cases	HRa (95 % CI)^b^	Cases	HRa (95 % CI)^b^
Women							
Morbidity	7307			4452		2855	
No health condition	5742	1.00	1.00	3407	1.00	2335	1.00
One health condition	1337	1.54 (1.46–1.64)	1.15 (1.08–1.22)	890	1.11 (1.03–1.19)	447	1.20 (1.09–1.33)
≥2 health conditions							
Low MDMS (first tertile, ≤22 points)	108	1.86 (1.54–2.25)	1.17 (0.97–1.42)	75	1.08 (0.86–1.36)	33	1.32 (0.94–1.86)
Medium MDMS (second tertile, >22 to ≤38 points)	93	1.73 (1.41–2.13)	1.22 (0.99–1.50)	64	1.14 (0.89–1.46)	29	1.29 (0.89–1.86)
High MDMS (third tertile, >38 points)	27	1.51 (1.04–2.21)	1.40 (0.96–2.04)	16	1.42 (0.87–2.32)	11	1.38 (0.76–2.49)
Previous SA							
No episodes	2855	1.00	1.00		–		–
1–4 episodes	4310	1.92 (1.91–1.94)	1.90 (1.88–1.92)		–		–
>4 episodes	142	2.33 (2.25–2.41)	2.29 (2.21–2.37)		–		–

^a^Crude hazard ratio and 95 % confidence interval

^b^HRa = hazard ratio and 95 % confidence interval adjusted for age and occupational social class

## Discussion

We created a new multidimensional multimorbidity score (i.e., the MDMS), using a methodology that allows us to combine chronic health conditions, health-related behaviors, and selected chronic symptoms. In contrast to previously developed multimorbidity measures which have been aimed to older and, typically, less healthy populations, our MDMS is more suitable for use in younger, and presumably healthier, working populations. We also found that the higher the multimorbidity score, the higher the risk of future SA episodes. This initial evaluation of its predictive ability suggests it can help identify people at risk, and thus prevent, delay, and/or mitigate the onset of future health conditions.

Regarding the composition of our MDMS, our findings revealed clinically logical relationships along two dimensions that may help inform the burden and distribution of multimorbidity beginning at an earlier point in adult life. The first dimension was conformed of highly related cardiovascular risk factors and health behaviors (seven in men and four in women). The second dimension grouped pain symptoms (i.e., in headache, neck, and back) and sleep disturbances, which are often associated with decreased self-perceived and mental health (Pikó et al. 1997; Ohayon 2002).

These results are in overall agreement with prior research on multimorbidity patterns in both working and patient populations (Holden et al. 2011; Prados-Torres et al. 2012). In older veteran primary care patients, a “metabolic” cluster was identified as being both the most prevalent and the one having the highest degree of relationship (Cornell et al. 2009); Similarly, both cardiovascular and chronic pain morbidity were identified as the most prevalent domains in primary care settings (Britt et al. 2008); Cardiovascular diseases, metabolic conditions and osteoarthritis are among the six most prevalent diseases within multimorbidity patterns (Violan et al. 2014). In an older German population, chronic low back pain and depression had the strongest association in clustering with other diseases (Schäfer et al. 2014); Moreover, mental health and musculoskeletal disorders tend to cluster together with pain symptoms, whereas substance and alcohol abuse cluster with both cardiovascular disease and mental health disorders (Prados-Torres et al. 2014), and a pain-related cluster, including migraine, neck, back, and other pains, was recently described in a working population (Holden et al. 2011). Although pain symptoms were available in our study, data on mental or musculoskeletal diseases were not and alcohol consumption did not load into any cluster, but this has already been reported by others (Holden et al. 2011).

Previous studies of multimorbidity usually define it as the co-occurrence of two or more diseases, without including behavioral risk factors or chronic symptoms that, as pointed out above, tend to naturally cluster with diseases. Thus, our outcome-independent, score-based approach has certain advantages, particularly in relation to typical multimorbidity research showing high variability of the multimorbidity prevalence estimates. Besides the contribution of methodological aspects (e.g., the type of population studied, data availability, sample size, recruitment strategy and data collection methods) (Fortin et al. 2012; Salive 2013), the high variability likely relates to the fact that the relative weight of a given chronic condition varies by specific outcomes, e.g., mortality or disease severity, leading to over or underestimation of the effect of some health conditions (Fortin et al. 2005). In contrast, and although more research is needed to better understand this variability, by not focusing on a specific outcome, we obtained a multimorbidity measure that can be used subsequently to assess its impact on several key health outcomes or target populations.

Computing a score may be useful for determining the burden and distribution of morbidity in any population of interest, and to identify profiles of individuals who might need special attention in terms of prevention strategies, medical care or health surveillance. Likewise, it may aid in projecting healthcare and other economic costs associated with populations having these characteristics. Traditionally, health care has focused on treatment of single diseases, without fully grasping the underlying patterns of coexisting diseases and other chronic conditions. Previous studies have shown the influence of multimorbidity on a wide range of outcomes (Smith et al. 2008; Schäfer et al. 2009; Aarts 2012), primarily in populations seeking health care. Whether this influence persists in presumably healthier persons, such as those in a working population, is less well known. Multimorbidity could also impact outcomes more relevant to occupational health, such as work ability or sickness absence. Thus, for example, we know that duration of sick leave can vary for a given condition, depending on gender, age, and presence of co-existing medical diagnoses (Ubalde-Lopez et al. 2013). Our multidimensional score was more sensitive in detecting a larger proportion of workers with single or multiple morbidities than including only chronic conditions.

Moreover, the MDMS obtained showed a predictive ability to detect an increased risk of incident SA episodes in both sexes, even after including prior SA episodes, already defined as a strong predictor for future SA, and adjusting for other co-variables. In our study, prior SA episodes were a strong predictor of future SA, more so than MDMS. This is not surprising, given that it is expected a history of SA would predict future SA (Roelen et al. 2011). However, the predictive effect of MDMS did not disappear after accounting for prior SA episodes. In fact, its effect was strongest among those without a prior SA episode, especially at high levels of MDMS, reflecting the added value of considering MDMS as a relevant indicator of future SA.

Although MDMS and incident SA were associated, a clear dose–effect relationship was not observed. This could be due, for instance, to a “threshold” effect, where the greatest association was observed at the highest tertile, but not in a stepwise fashion, since the risk for the lower tertiles appears to be similar. Further research is needed to better elucidate the dose–response relationship between MDMS and SA as well as other outcomes.

To date, morbidity indices have usually been created to predict specific outcomes (e.g., mortality rates, hospitalization indices or physical impairment) by including severity-weighted conditions, typically based on the prevalence of selected health conditions in a specific population. Being based on a specific index-outcome relation they can only, by definition, be assumed to have predictive effects on that specific outcome. As such, these indices are of limited generalizability. In contrast, our proposed MDM indicator goes beyond simply measuring the presence of certain health conditions and applying weights based on the sample-specific prevalence. The MDMS is based on the non-random relationship among health-related conditions, available in any population, independent of a pre-specified outcome. It can therefore be generated in any given population and may be useful to test predictive effects on a variety of outcomes.

Regarding the use of MCA in our approach, prior studies have used different statistical strategies to reduce long lists of clinical variables, in order to identify multimorbidity patterns (Britt et al. 2008; Cornell et al. 2009; Holden et al. 2011; Prados-Torres et al. 2012). Most have relied on factor analysis, which is best used when the variables are either continuous or semiquantitative (e.g., Likert-type responses). MCA is better suited to examining relationships among categorical variables, whether nominal or discrete, allowing the identification of clusters by reducing data dimensions, independently of the outcome. Given that all our questionnaire items had categorical responses, using MCA was more appropriate, despite having only been used sparingly in the study of multimorbidity (García-Olmos et al. 2012).

Multimorbidity prevalence by sex has also varied in previous studies, as it did in our study, depending on the age group and target population, with inconsistent findings, including no difference or a higher prevalence in either sex. In our population, women attended fewer medical evaluations in 2006 than men, which might explain their underrepresentation in the study. There are a couple of plausible explanations for this. Women, especially in the younger age range of a working population, are more likely to get routine health evaluations through their primary care provider or gynecologist, than through a physician selected by a health insurance company (Case and Paxson 2005; Carretero et al. 2014). On the other hand, in our study population, men were more likely to be employed in manual occupations, whereas women were in administrative/clerical jobs. The nature of occupational risks, therefore, is likely to have been different, which may have led to a larger proportion of men undergoing what was perceived as a work-related health evaluation. Thus, some degree of underestimation of multimorbidity prevalence among women in this study has to be considered. Nevertheless, our results highlight the need to analyze health-related outcomes separately for men and women.

Although morbidity is well known to increase with age, it is not just an issue of the elderly, but also worth considering in younger groups, since patterns of risk factors, health conditions, and multimorbidity prevalence can vary along the life course as well as within age groups (Taylor et al. 2010; Prados-Torres et al. 2012). It is reasonable to consider multimorbidity as a dynamic phenomenon that evolves over time. In both women and men, at early ages, there are likely risk factors and behaviors that predispose to the development of chronic disease in the middle part of life. And in later stages, complications from these diseases can take on a dominant role, at a high cost to both individuals and society. For many cardiovascular and metabolic diseases, these patterns are well established, e.g., the effect of hypercholesterolemia and smoking in coronary artery disease, or obesity in diabetes (Prados-Torres et al. 2012). Risk factors are considered the starting point for future morbidity, but the decision to include them in multimorbidity measures is admittedly controversial (Guthrie et al. 2012). Although it is unclear whether risk factors impact how diseases cluster, a recent study found obesity as a predictor of multimorbidity to have a role in the clustering of chronic conditions (Agborsangaya et al. 2013). The expected increase in major risk factors (Vaughan-Jones 2009), strongly associated with unfavorable health states and a higher number of chronic health conditions (Surís et al. 2008; WHO 2009), points to the need for their inclusion in the concept of multimorbidity, at least in younger, healthier populations. By identifying these at-risk groups earlier, interventions could be designed to promote healthier habits which could impact future SA, among other outcomes.

Our results should be interpreted taking into account certain limitations and considerations. First, although our study population was highly dominated by men, which limits the comparison of multimorbidity profiles between sexes, the distribution of this insured group was comparable to that of the general Spanish workforce (data not shown). Second, given that participation in the 2006 medical evaluation was voluntary, some degree of selection bias may have affected our findings. Third, generalization to the non-working or even the entire working population should be done with caution since working populations, such as ours, and especially those undergoing a health examination, tend to be healthier and possibly more motivated to know their health status than the general or other populations (Li and Sung 1999; Loeppke et al. 2010). Future research should be conducted with different datasets to both replicate our findings and provide evidence for the generalizability of the proposed MDMS score. However, the working population was appropriate for our study aim and our findings are, at a minimum, likely to be relevant to other working populations. Indices of comorbidity, such as the Charlson Index, have proven useful as a tool for interventions at the individual clinical level (D’Hoore et al. 1996). In contrast, the MDMS might be more appropriate for predicting health-related outcomes at the population level. Although our results may have clinical significance, given the early stage of our research, it is premature to assert how clinically useful the MDMS would be.

Fourth, our data came from only one public health insurance company, but this was one of the largest in the country with representation throughout Spain, where healthcare coverage is comparable to other western European Union countries (Figueras et al. 2008). Fifth, being a working population, our participants were relatively younger than the overall population, and their morbidities are likely to fluctuate over time. Our study design had only a baseline measurement of multimorbidity, which precluded examining temporal changes in the relationship between MDMS and SA, which would be better addressed using a repeated measures design.

Other limitations relate to the use of questionnaire-based self-reported data. The data had been previously collected, but were not specifically designed to study multimorbidity. Thus, we may have underestimated the prevalence of health conditions, behaviors, and other morbidities beyond those included in this study. For example, most of the available information centered on cardiovascular factors, and data on other common health conditions (e.g., mental, musculoskeletal disorders or cancer) were not collected (Diederichs et al. 2011). The number of health conditions included in previous studies of multimorbidity has varied widely (Huntley et al. 2012) and will always represent a limitation. Nonetheless, by including symptoms (e.g., pain and sleep disturbances) as a dimension of disease, some of these other pathologies may have been indirectly captured. The chronic conditions were identified by self-report, but not confirmed clinically (e.g., physician diagnosis) (Preen et al. 2004). However, the predictive accuracy of self-reported morbidity as the basis for an index of chronic conditions has been previously validated in health interview surveys (Rius et al. 2008).

Finally, we must consider that multimorbidity occurs within a multifactorial context determined by individual, behavioral, social, cultural, economic, and environmental circumstances (Salive 2013). Thus, there are likely to be complex interactions underlying the clustering of coexisting health conditions that go beyond simple biological factors, so a better understanding of the etiology, underlying pathogenesis and patterns of multimorbidity is key to advancing research in this field (Starfield 2011b). The study of multimorbidity is relatively recent, and greater consensus on its measurement and interpretation is needed. To enhance generalizability, future studies should validate current approaches to its measurement, so that it can be applied in different settings and populations. In contrast with patient and/or older populations, studies on the applicability of multimorbidity indicators to younger and healthier populations are scarce. Here we propose what may be a more holistic approach to examining multimorbidity, based on calculation of a score rather than estimating prevalence, incorporating factors not limited to chronic diseases, and applying it earlier in the life course. Although our MDMs ended up including conditions that are not only relevant to young but also older populations, this does not diminish the potential utility of the strategy proposed to create the multimorbidity score.

Next steps will include examining this approach in other populations and determining its value by examining the degree to which it is able to predict various outcomes. These outcomes should not be limited to indicators such as mortality or disease severity and their burden on the healthcare system, but also include the impact of multimorbidity as a potential determinant of ability to work, sick leave, or quality of life.

## Electronic supplementary material

Below is the link to the electronic supplementary material.

Supplementary material 1 (DOCX 14 kb)

Supplementary material 2 (DOCX 13 kb)
