# Supplementary material for: Measuring multimorbidity in a working population: the effect on incident sickness absence
Source: Int Arch Occup Environ Health. 2015 Nov 28;89:667–78. doi: 10.1007/s00420-015-1104-4 (PMC4828479; doi:10.1007/s00420-015-1104-4)
Supplement: Supplementary file 1 — Supplementary material 1 (DOCX 14 kb) [file 420_2015_1104_MOESM1_ESM.docx]

| Web Appendix **1.** Prevalence of chronic conditions, Body Mass Index, and health behaviors among a population of Spanish workers who underwent a medical evaluation during 2006. | | | | | |
| --- | --- | --- | --- | --- | --- |
|  |  |  |  |  |  |
| **Characteristics** | | **MEN** | | **WOMEN** | |
|  |  | N | % | N | % |
| **Chronic conditions** | |  |  |  |  |
|  | None | 178,815 | 66.5 | 66,297 | 64.2 |
|  | Hypertension | 18,025 | 6.7 | 3,268 | 3.2 |
|  | Hyperlipidemia | 22,280 | 8.3 | 4,615 | 4.5 |
|  | Diabetes | 5,074 | 1.9 | 426 | 0.6 |
|  | Venous thrombosis | 279 | 0.1 | 90 | 0.1 |
|  | Coronary artery disease | 1,406 | 0.5 | 127 | 0.1 |
|  | Cerebrovascular disease | 316 | 0.1 | 85 | 0.1 |
|  | Peripheral vascular disease | 179 | 0.1 | 37 | 0.0 |
|  | Missing | 37,636 | 10.1 | 27,677 | 10.3 |
| **Body Mass Index (BMI)** | |  |  |  |  |
|  | Underweight (<20) | 7,942 | 2.9 | 14,717 | 14.3 |
|  | Normal weight (20-29) | 208,884 | 77.6 | 78,042 | 75.6 |
|  | Overweight/Obesity (>=30) | 49,840 | 18.5 | 9,193 | 8.9 |
|  | Missing | 2,418 | 0.9 | 1,335 | 1.3 |
| **Tobacco consumption** | |  |  |  |  |
|  | Never smoke | 66,468 | 24.7 | 34,344 | 33.3 |
|  | Current smoker | 119,567 | 44.4 | 38,558 | 37.3 |
|  | Former smoker | 48,022 | 17.8 | 16,114 | 15.6 |
|  | Missing | 35,026 | 13.0 | 14,271 | 13.8 |
| **Alcohol consumption** | |  |  |  |  |
|  | Never drinker | 75,963 | 28.2 | 55,145 | 53.4 |
|  | Occasional (<once/week) | 69,444 | 25.8 | 27,399 | 26.5 |
|  | Weekend | 65,320 | 24.3 | 15,332 | 14.8 |
|  | Daily moderate (<140gr/week) | 53,092 | 19.7 | 4,251 | 4.1 |
|  | Daily high (>140gr/week) | 519 | 0.2 | 2 | 0.0 |
|  | Former drinker | 457 | 0.2 | 23 | 0.0 |
|  | Missing | 4,288 | 1.6 | 1,135 | 1.1 |
| **Total** | | 269,083 | 100 | 103,287 | 100 |
