# Supplementary material for: Measuring multimorbidity in a working population: the effect on incident sickness absence
Source: Int Arch Occup Environ Health. 2015 Nov 28;89:667–78. doi: 10.1007/s00420-015-1104-4 (PMC4828479; doi:10.1007/s00420-015-1104-4)
Supplement: Supplementary file 2 — Supplementary material 2 (DOCX 13 kb) [file 420_2015_1104_MOESM2_ESM.docx]

| Web Appendix **2.** Distribution of symptoms in a population of Spanish workers who underwent a medical evaluation in 2006. | | | | |
| --- | --- | --- | --- | --- |
|  |  |  |  |  |
| **Symptoms** | **MEN** (N=269,083) | | **WOMEN** (N= 103,287) | |
|  | N | % | N | % |
| Fatigue | 3,951 | 10.4 | 4,564 | 17.1 |
| Dizziness | 117 | 0.3 | 108 | 0.4 |
| Sweats | 85 | 0.2 | 53 | 0.2 |
| Itching | 6 | 0.0 | 4 | 0.0 |
| Weight loss | 63 | 0.2 | 45 | 0.2 |
| Feeling feverish | 58 | 0.2 | 30 | 0.1 |
| Chest tightness | 143 | 0.4 | 92 | 0.3 |
| Cough | 193 | 0.5 | 72 | 0.3 |
| Headache | 1,050 | 2.8 | 1,243 | 4.6 |
| Low back pain | 4,923 | 12.9 | 2,455 | 9.2 |
| Heartburn | 1,099 | 2.9 | 297 | 1.1 |
| Urinate pain | 88 | 0.2 | 54 | 0.2 |
| Menstrual symptoms | - | - | 246 | 0.9 |
| Lack of appetite | 70 | 0.2 | 84 | 0.3 |
| Breathlessness | 304 | 0.8 | 201 | 0.8 |
| Feeling sick | 49 | 0.1 | 48 | 0.2 |
| Sleepiness | 195 | 0.5 | 196 | 0.8 |
| Dyspnea | 105 | 0.3 | 37 | 0.1 |
| Neck pain | 2,399 | 6.3 | 3,270 | 12.2 |
| Upper respiratory tract | 3,799 | 10.0 | 1,454 | 5.4 |
| Lower respiratory tract | 81 | 0.2 | 28 | 0.1 |
| Sleep disturbances |  |  |  |  |
| During the night | 15,893 | 41.8 | 10,748 | 40.1 |
| Early in the morning | 3,395 | 8.9 | 1,444 | 5.4 |
| **Total** | 38,066 | 100 | 26,773 | 100 |
